# Modern Sociology

**Published:** 1900-12-29

**Authors:** 


### Modern Sociology.

### SODA-WATER BOTTLES.

That a bottle of soda water is a possible weapon of offence we all know, and it is easy to understand that some danger must be associated with the filling of the bottles with the aerated liquid. But, indeed, the danger begins before the bottle reaches the soda-water factory at all. After annealing, and before sending out, many manufacturers of bottles test them by filling them with compressed air. In a work visited by the Committee the bottles were tested at a pressure of 150 lbs. It is creditable to the glass manufactures that they should be careful that no imperfect bottle should be sent out, but it is obvious that the testing of the bottles may be attended with some danger to those who perform the task. The danger is perhaps greater than that incurred by the actual bottlers, who, after all, are dealing with bottles which have successfully undergone the test.

Both in testing and in filling the bottles they are placed on a machine, where they are filled with, according to the work in process, compressed air, or carbonic acid gas. The latter is the gas by which our mineral waters are aerated. It is generated from a combination of sulphuric acid and chalk or whiting, and in the process of preparing the gas the committee saw no risk or danger to the workers. Sometimes the gas is introduced into the water before the bottle is filled, but oftener the bottle is filled with pure water first, and the gas is pumped in afterwards. When the bottles are closed with the patent glass ball stopper, the ball is inside the bottle and is forced to the neck by the pressure of the gas. Other bottles have to be corked and wired after being taken from the machine. Sometimes bottles break at this stage, or when being moved about after the corking is finished, but the chief danger is that the bottles will burst when they are being filled with the gas. So common is this that at one factory of no great size visited by the Committee six bottles "flew" within ten minutes, and this on a day in December, when neither excessive heat nor pressure of work could be regarded as accountable for the accidents. It has been found by experience that the green glass seen in seltzer-water bottles gives a more painful cut than that inflicted by the common white glass of soda-water bottles.

The bottling and testing machines are fenced at each end with wire gauze, and the workers also wear wire gauze protectors of various kinds. But a great deal of latitude is allowed in the fashion of these, and in some of the glass works where the bottles are tested the workers have no protectors at all. The Committee recommend that the wearing of protectors should be made compulsory. A complete outfit consists of a face-guard and gauntlets. Sometimes the gauntlet is worn only on one arm, or wire spectacles take the place of the face-guard. In some factories the bottlers wear some kind of protector, but none is supplied for the wirers and labellers, although, as the Report says: "The liability to burst does not become so small after filling as to warrant either wirers or labellers to dispense with careful precautions." The gauntlet may be like a mitten, coming down to the finger joints, but many of the labellers prefer a shorter armlet, that reaches only to the wrist, in order to have their fingers free. But the Committee saw labellers working with their hands fully protected and seeming to find no inconvenience from the long gauntlet, and they are of opinion that the wearing of full-length gauntlets on both arms should be made universal and compulsory for bottlers sighters, and labellers alike. "Sighters" are a class employed to inspect the bottles or siphons before they leave the factory, in order to see that they are perfectly clean and pure. At present they are furnished with no protection whatever. For the wirers, the Committee think that it might be sufficient to wear a full-length gauntlet on one arm and a shorter one on the other. They think that all workers should wear face-guards, masks, or veils of wire gauze, and recommend the still more complete fencing of the bottling machines. In one factory the workers are made to pay half the cost of any protectors they use, in order, it was explained, to make them take care of them. Yet in factories where the employer supplied the masks and gauntlets the Committee did not find the carelessness in using them which was made the excuse for this tax.

In the progress of their inquiries into the conditions of labour in soda-water factories, the attention of the Committee was drawn to the process of labelling, a matter which they afterwards investigated in connection with thread-works. With regard to the mineral-water labels, they found nothing to object to in England, where the work is done by women, who dab the label on a quantity of paste which is on a board by their side, and then stick it on the bottle. But in Ireland the work is mostly done by boys, who lick with their tongues labels which are already gummed. They put several labels in their mouth at once, and it is said, "take them out and stick them on with amazing rapidity and deftness." One witness informed the Committee that "he had labelled 50 dozen in a day in this manner. He habitually did over 40 dozen." Even this astonishing record is beaten by some of the women and girls who lick and put on labels in the thread mills. In one factory in Lancashire there were employed at the time of the Committee's visit "some twelve full-timers, who each licked from 40 to 50 gross labels per day, and thirty-five half-timers, who accomplished from 20 to 25 gross per day." One woman informed the Committee that when busy she could complete 45 gross of bobbins a day, or allowing a ticket for each end of the bobbin, 90 gross of labels a day! Dr. Oliver, the medical member of the Committee, expresses the opinion that since the work is usually done by young persons and children at an age when growth is active and the system requires all its digestive secretions, the daily loss of saliva to the system cannot but be prejudicial to health. Dr. Oliver's opinion is borne out by that of Dr. Gibb, a Paisley doctor. Paisley is a prominent centre of the thread industry. Dr. Gibb had seen a great deal of factory hands, and says on this point that girls who had been employed for a somewhat lengthened period at ticketting on the old tongue-licking system have suffered most seriously from gastric disturbances in the form of dyspepsia and actual inflammation of the organ. He thinks that these disorders are due to the bad effects of what he frankly calls "this wholly unnecessary and—I should say—rather disgusting practice." Messrs. Coats, of Paisley, use a damper for this work, and many other firms are now obtaining these dampers from them. As the colours of the labels often contain copper and lead, though only in small quantities, there are even stronger reasons than the lessening of the salival secretion for stopping the practice of licking labels, and there is every prospect of the practice soon coming to an end.

